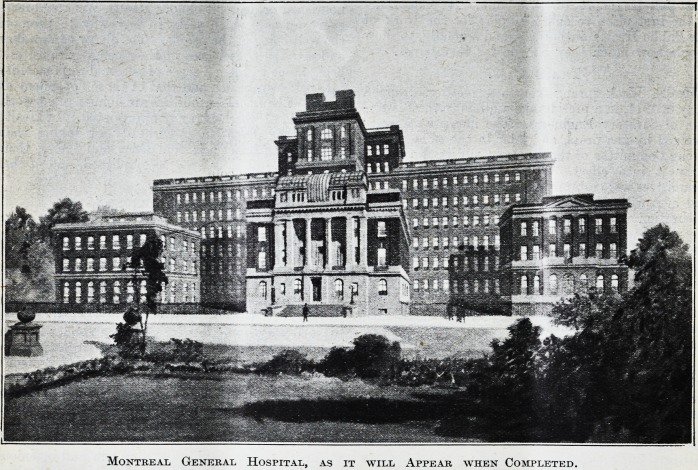# Hospital Problems in Canada: Work of 1923

**Published:** 1924-03

**Authors:** 


### HOSPITAL PROBLEMS IN CANADA.

### THE WORK OF 1923.

Dr. MacEarchern, President of the American Hospital Association, writing in the *Modern Hospital*, reports that "a greater interest in hospital problems in the Canadian field has been evidenced on all sides during 1923. There is an ever-growing realisation that hospital facilities are essential to every community and should be available to provide as broad a service as that community requires."

### Beds and Population.

The average rate of beds to population for civilians in Canada is 1 to 347. Authorities are generally agreed that there should be five to seven beds per thousand population for general hospital purposes. In the United States and Canada we find that one out of every ten or eleven persons needs hospital service at some time or other during the year. In Canada, therefore, in 1923, on this basis of calculation, approximately 878,848 persons would require hospital care. There are 25,826 general hospital beds available in Canada. Each bed, on the average, cannot take care of more than twenty patients annually. Therefore, the present available accommodation can only provide for 516,520 patients. The remainder must be treated outside the hospital.

### Hospitals and the Middle Class.

Many persons cannot afford hospital service to-day because of the cost. The hospital very largely really serves only two classes—the poor, or the charity patient, and the comparatively rich, the former because the State so regulates and provides therefor, the latter because they can pay; whereas the largest group, or the so-called middle class, who cannot accept charity on the one hand or pay the pecuniary charges on the other, must make other arrangements, and are usually cared for in their own homes. This large and important class is made up of honest, industrious people of limited means whose resources are heavily taxed in building up homes and raising families, and who, therefore, are obliged to refrain from contracting additional expenses. Not infrequently, however, the illness is of such a nature that treatment should be administered under the most favourable hospital conditions. This problem needs very serious consideration.

Montreal General Hospital, as it will Appear when Completed.

### The Problem of Rural Districts.

The distribution of hospitals throughout rural districts should receive close study. Available hospital service of as wide a range as advisable is necessary in every community covering a reasonable area with a sufficient population. These rural or community hospitals in the outlying parts should at least give a service to emergency cases that cannot be transported, maternity and medical cases. If the case can be safely transported, I believe that major surgery should always be taken to the large hospital centre with its more elaborately organised facilities and personnel of specialists.

LACK OF INSPECTION.

In many of the provinces of Canada hospital inspection is lacking. We need systematic and competent inspection, which can only be made by persons well experienced and tried in all the branches of hospital administration. One of the greatest factors for the development of the hospital system of Canada would be the establishment of competent, instructive and constructive inspection of our institutions, with a department of information on hospital matters. There is always, of course, the danger of "political inspection" rather than the kind which I would like to term "service inspection."

HOSPITAL ASSOCIATIONS.

There are at present four hospital associations in Canada, and these are found in the four western provinces. They are all active and growing, each holding an annual conference of two or three days' duration, from which has arisen the greatest benefit to all the hospitals in the particular community. The most noticeable feature of the meetings this year has been the attendance of trustees in every instance in much larger number than in former years. They took an active part in all proceedings. Progress has been made in all branches of nursing, and is evidenced particularly in a better type of young women applying, an increased number, improved teaching facilities, and decidedly more satisfactory living conditions. Public health nursing courses in our universities have been fairly well patronised during the year.

## Figures and Tables

**Figure f1:**